# Preliminary Free Energy Map of Prebiotic Compounds Formed from CO_2_, H_2_ and H_2_S

**DOI:** 10.3390/life12111763

**Published:** 2022-11-02

**Authors:** Jeremy Kua, Nicole A. Miller

**Affiliations:** Department of Chemistry & Biochemistry, University of San Diego, San Diego, CA 92110, USA

**Keywords:** origin of life, thermodynamics, prebiotic chemistry, sulfur, sugars

## Abstract

What kinds of CHOS compounds might be formed in a prebiotic milieu by reducing CO_2_ in the presence of H_2_ and H_2_S? How might the presence of sulfur influence the chemical composition of the mixture? We explore these questions by using first-principles quantum chemistry to calculate the free energies of CHOS compounds in aqueous solution, by first generating a thermodynamic map of one- and two-carbon species. We find that while thiols are thermodynamically favored, thioesters, thioacids, and thiones are less favorable than their non-sulfur counterparts. We then focus on the key role played by mercaptoacetaldehyde in sulfur analogs of the autocatalytic formose reaction, whereby the thiol group introduces asymmetry and potential thermodynamic selectivity of some compounds over others.

## 1. Introduction

Sulfur has been implicated in origin-of-life scenarios, from the discovery of chemotrophic organisms at hydrothermal vents and the proposal of pyrite surfaces [1] as a driving force for prebiotic metabolism, to De Duve’s thioester world [2] and more recent work invoking a cyanosulfidic world [3] as prebiotic milieus. The core of extant metabolism [4] consists of a small subset of molecules containing the elements carbon, hydrogen, and oxygen. Sulfur participates in the form of Coenzyme A at specific junctures, embedded in a system that is overall highly regulated by enzymes and co-factors. Sulfur also participates through amino acids in extant life; see Youssef-Saliba and Vallée for a recent review in their role and significance in prebiotic chemistry [5].

Energy is at the heart of metabolism in extant life, and we expect it to be the driving force in the dynamic creation of proto-metabolic chemical systems. Our most recent study presented first-principles quantum chemical calculations of the relative free energies in aqueous solution of a wide range of potential CHO-containing metabolites [6]. In that scenario, sulfur was not included, and the free energies of CHO compounds were calculated with reference to carbon dioxide (specifically H_2_CO_3_ as the aqueous form) and molecular hydrogen as the reducing agent. In that work, we zeroed in on the smallest potential autocatalytic cycle that utilized a C_1_ molecule as the “food” source, and a linchpin C_2_ molecule that is regenerated in a three-step reaction cycle: C_2_ + C_1_ → C_3_, C_3_ + C_1_ → C_4_, and C_4_ → C_2_ + C_2_. This smallest cycle is present in the autocatalytic formose reaction [7], amidst a mess of larger cycles and side reactions. The result is a complex and messy mixture with a large array of compounds [8]. A recent excellent review on the formose reaction in prebiotic chemistry including issues of chirality has been written by Martinez et al. [9].

Our present study treads similar ground, but includes the addition of H_2_S as both reactant and reductant; H_2_S is the source of incorporating sulfur in the formation of CHOS compounds. To get a lay of the land, we first calculate the relative free energies of the C_1_ and C_2_ CHOS compounds that are potentially formed, and compare these to the CHO compounds from our previous work. We find that thiols are thermodynamically favored over their alcohol counterparts, whereas thiones (C=S) are disfavored relative to carbonyl (C=O) groups. (We use the nomenclature for sulfur compounds provided by Toohey and Cooper [10]). Thiocarboxylic acids (both the carbothioic *O*-acid and *S*-acid) are also disfavored relative to carboxylic acids; we will refer to these as the thione-acid and the thioacid, respectively, for short. In examining the thermodynamics of coupling reactions that form C-S bonds, we find that formation of dialkylsulfides is exergonic, while condensation reactions to form thioesters are endergonic.

This paper then focuses on investigating both the thermodynamics and kinetics of the simplest formose-like autocatalytic cycle for CHOS compounds, comparing our free energy map to our previous calculations on the CHO system [11]. The inclusion of sulfur in the system increases the number of possible chemical species in the mixture. Furthermore, the asymmetry provided by having thiol groups leads to energetic differences favoring some compounds over others. In a sense, the thiol acts as a “directing” group that influences both the kinetics and thermodynamics of the aldol-like reactions in this system. We also analyze how sulfur influences the disproportionation (Cannizzaro) reactions that give rise to a range of compounds with different oxidation states. These “side” reactions (which are arguably critical towards generating a diverse mixture) are a significant part of the messy formose reaction [8].

While thioesters are of interest in the construction of proto-metabolic cycles, they are not a focus of this paper. The present work on the CHOS analog to autocatalytic cycles in the formose reaction sets the stage for exploring the role of thioesters in such cycles, and we are actively generating data that will be presented in a follow-up paper. The present paper is structured as follows. After describing our computational methods, the combined results and discussion section (Section 3) will cover (1) formation of C_1_ CHOS compounds, (2) a survey of the energy landscape of C_2_ CHOS compounds, (3) a discussion of C-S bond forming reactions, (4) our detailed examination of the CHOS analog of the formose reaction, (5) investigating if thiol groups can influence the selection of some sugars over others, and (6) products formed in the CHOS analog to the Cannizzaro reaction.

## 2. Materials and Methods

Since we will be comparing our calculations on the CHOS system to our previous work on the CHO system, we use the same computational protocol found in those papers [6,11]. We provide herein a brief description of that protocol for the convenience of our readers. Some of the text in this section is reproduced from our most recent work [6] since we think our previous description is both clear and succinct. Essentially, we calculate the free energies using quantum chemical methods, and our protocol shows good agreement with available experimental results for CHO systems [11,12,13]. Here, are the computational details:

The structure of each molecule is optimized and its electronic energy calculated at the B3LYP [14,15,16,17] flavor of density functional theory with the 6-311G** basis set. To maximize the probability of finding global minima, multiple conformers are generated using molecular mechanics (MMFFs force field [18]). The optimized structures are embedded in a Poisson-Boltzmann continuum to calculate the aqueous solvation contribution to the free energy. While this does not provide a specific concentration, it assumes a dilute solution such that the electrostatic field generated by a neighboring solute molecule is effectively screened by the water solvent. One can consider all solutes to have the same relative concentrations in our calculations. Zero-point energy corrections are included, and we apply the standard temperature-dependent enthalpy correction term (for 298.15 K) from statistical mechanics by assuming translational and rotational corrections are a constant times *kT*, and that low frequency vibrational modes generally cancel out when calculating enthalpy differences. So far, this is standard fare.

However, entropic corrections in aqueous solution are more problematic [19,20,21]. Changes in free energy terms for translation and rotation are poorly defined in solution due to restricted complex motion, particularly as the size of the molecule increases (thus increasing its conformational entropy). Free energy corrections come from two different sources: thermal corrections and implicit solvent. Neither of these parameters is easily separable, nor do they constitute all the required parts of the free energy. We follow the approach of Deubel & Lau [22], assigning the solvation entropy of each species as *half* its gas-phase entropy (calculated using standard statistical mechanics approximations similar to the enthalpy calculations described above), based on proposals by Wertz [23] and Abraham [24] that upon dissolving in water, molecules lose a constant fraction (~0.5) of their entropy.

To estimate activation energies, transition states were optimized by including several explicit water molecules (two is usually optimal, but occasionally one or three give lower barriers) to aid proton transfer. All calculated transition states have one significant negative eigenvalue corresponding to the reaction coordinate (eigenvector) involving bond breaking/forming. Several conformers are tested in each case and we only report the lowest calculated barriers.

When put to the test by first calculating the equilibrium concentrations in a self-oligomerizing solution of 1 M glycolaldehyde at 298 K, our protocol fared very well compared to subsequent NMR measurements [13]. Our relative Gibbs free energies in aqueous solution are typically within 0.5 kcal/mol compared to experiment. That being said, our protocol did show systematic errors of 2–3 kcal/mol when calculating barriers and comparing to experimental results. Going to a higher level of theory does not reduce this error [25]. This may seem surprising but quantum chemistry is about error cancellation, and our protocol (with its foibles, including the simplistic entropy correction) has worked well even with this systematic error for activation barriers. Thus, we do well on thermodynamics and just okay on kinetics (but at least we’re in the ballpark).

The *relative* aqueous Gibbs free energies, designated *G_r_*_0_, are calculated with respect to the reference molecules: CO_2_, H_2_, H_2_S and H_2_O. These molecules are assigned *G_r_*_0_ = 0.0 kcal/mol. (Note that in our most recent work, we used H_2_CO_3_ instead of CO_2_ as the reference to directly compare with experimentally derived thermodynamic data from Alberty [26], but since this same experimental data is not available for the sulfur-compounds, it is “cleaner” to use CO_2_ as the reference.) Our reported *G_r_*_0_ values are for the lowest energy conformer of each structure. Assigning reference molecules allows us to quickly compare energies among various compounds. For a chemical reaction, the difference in free energies will be designated Δ*G*, calculated as *G_r_*_0_(products) − *G_r_*_0_(reactants). Sample calculations will be shown in the first part of the Results and Discussion section (Section 3).

## 3. Results and Discussion

### 3.1. Formation of C_1_ Compounds and Choosing Reference States

With the reference molecules (CO_2_, H_2_, H_2_S and H_2_O) assigned *G_r_*_0_ = 0.0 kcal/mol, we can determine *G_r_*_0_ values for CHOS species by calculating Δ*G* for the formation reaction of each compound where the carbon “food” source is CO_2_, the sulfur source is H_2_S, additional source of hydrogen as a reductant comes from H_2_, and H_2_O is a byproduct of the reduction reaction. We group compounds by oxidation number of carbon, formally calculated by assigning the oxidation numbers of H (+1), O(−2) and S(−2). Thus, in CO_2_, carbon has oxidation number +4. If CO_2_ is fully reduced to methane (carbon in −4 oxidation state),

CO_2_ + 4 H_2_ → CH_4_ + 2 H_2_O   Δ*G* = –39.5 kcal

and therefore, *G_r_*_0_ of CH_4_ is −39.5 kcal/mol. If CO_2_ is not fully reduced, there are several possibilities.

(a) Carbon in −2 oxidation state:

CO_2_ + 3 H_2_ → CH_3_OH + H_2_O     Δ*G* = –11.2 kcal

CO_2_ + H_2_S + 2 H_2_ → CH_3_SH + 2 H_2_O   Δ*G* = –19.2 kcal

Thus, *G_r_*_0_ of CH_3_OH and CH_3_SH are −11.2 and −19.2 kcal/mol respectively. Both reactions are exergonic. The thiol is more stable than the alcohol by 8 kcal/mol. In a prebiotic environment where H_2_S is present to reduce CO_2_, we expect to observe methanethiol. The relative amount of H_2_S versus H_2_ would lead to different relative ratios of CH_3_SH and CH_3_OH in the product mixture (There would of course be many other compounds!).

(b) Carbon in zero oxidation state:

CO_2_ + 2 H_2_ → CH_2_O + H_2_O     Δ*G* = +7.9 kcal

CO_2_ + H_2_S + H_2_ → CH_2_S + 2 H_2_O   Δ*G* = +21.2 kcal

Thus, *G_r_*_0_ of CH_2_O and CH_2_S are +7.9 and +21.2 kcal/mol respectively. Both reactions are endergonic, i.e., it is thermodynamically unfavorable to form formaldehyde and its thione counterpart by reducing CO_2_. Forming CH_2_S with its weaker C=S double bond is significantly unfavorable. However, in aqueous solution, hydration can take place across the double bond.

CO_2_ + 2 H_2_ → CH_2_(OH)_2_         Δ*G* = +3.3 kcal

CO_2_ + H_2_S + H_2_ → CH_2_(SH)(OH) + H_2_O   Δ*G* = +4.0 kcal

For the hydrates, the sulfur-containing compound is marginally less stable (by 0.7 kcal/mol) than its counterpart. Both hydrates are still slightly higher in free energy compared to the reactants, but are now likely to be accessible.

(c) Carbon in +2 oxidation state:

CO_2_ + H_2_ → H(C=O)OH       Δ*G* = +2.7 kcal

CO_2_ + H_2_S + H_2_ → H(C=O)SH + H_2_O   Δ*G* = +12.8 kcal

CO_2_ + H_2_S + H_2_ → H(C=S)OH + H_2_O   Δ*G* = +15.4 kcal

CO_2_ + 2 H_2_S → H(C=S)SH + H_2_O    Δ*G* = +18.6 kcal

While formation of formic acid is only marginally endergonic from CO_2_ reduction, all three sulfur analogs are significantly higher in free energy. The thioacid, the best of the three, is 10 kcal/mol less stable than its carboxylic acid counterpart. (The two thione acids are even less stable.) This suggests that if a thioacid can be formed in some way, its hydrolysis to the carboxylic acid would be 10 kcal downhill and can be utilized to drive an uphill C–C bond-forming carboxylation reaction, typically 4–8 kcal endergonic based on our previous work. (We will see in a later section that thioesters are typically 6–7 kcal uphill from their hydrolyzed product.)

In prebiotic experiments for carbon fixation, COS has been used as an activating reagent (and the carbon source). The reaction CO_2_ + H_2_S → COS + H_2_O is endergonic by 10.5 kcal. Thus, we can assign *G_r_*_0_ of COS as +10.5 kcal/mol. Carbon monoxide has also been used as an activated reactant in prebiotic chemistry. The reaction CO_2_ + H_2_ → CO + H_2_O is endergonic by 11.3 kcal. Thus, we assign *G_r_*_0_ of CO as +11.3 kcal/mol. If either COS or CO are used as the carbon source rather than CO_2_, the formation of formaldehyde, its hydrate, or CH_2_(SH)(OH) are now exergonic reactions. Formic acid is also downhill ~8 kcal/mol, and the thioacid is now only marginally higher in energy (than COS or CO) and likely to be accessible.

**Figure 1:**
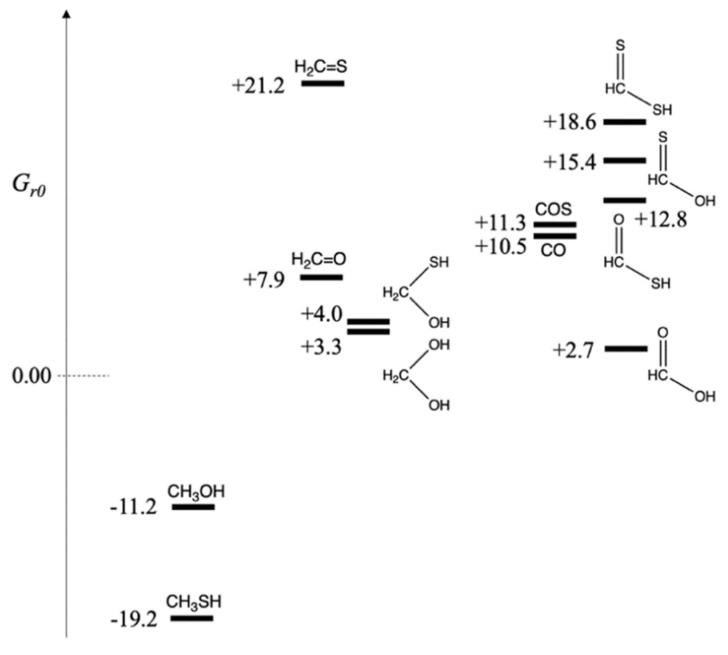
Free Energy Map of C_1_ CHOS compounds (*G_r_*_0_ values in kcal/mol).

The relative free energies of the possible C_1_ compounds are shown in Figure 1, grouped by oxidation state of carbon. On the left are the more reduced compounds, CH_3_SH and CH_3_OH with oxidation state of −2. In the center are CH_2_O, CH_2_S, and their hydrates at zero oxidation state. Furthermore, on the right are the acids with oxidation state +2. Carbon monoxide (the dehydrate of formic acid) is in this group, and because of the similar prebiotic chemistry of COS and CO, we have grouped them together. While we have formally assigned sulfur an oxidation number of −2 (so it can be grouped alongside oxygen for ease of analyzing the results), the electronegativity of sulfur is not too different from carbon. Our formal assignments are a bookkeeping method for ease of presentation, allowing us to group together compounds that only differ by swapping an S with an O or vice versa. 

Figure 1 makes it clear which compounds are accessible downstream using CO or COS as the carbon source rather than CO_2_. The fact that both CO and COS are ~11 kcal/mol less stable in free energy than CO_2_ allows them to function as activated reactants and drive subsequent reactions along a downhill thermodynamic gradient. However, if only CO_2_ was available as the carbon ‘food’ source, it is less likely that thioacids or thiones would be accessible; and the main C_1_ sulfur-containing compound would be CH_3_SH.

### 3.2. The Free Energy Landscape of C_2_ Compounds

**Figure 2:**
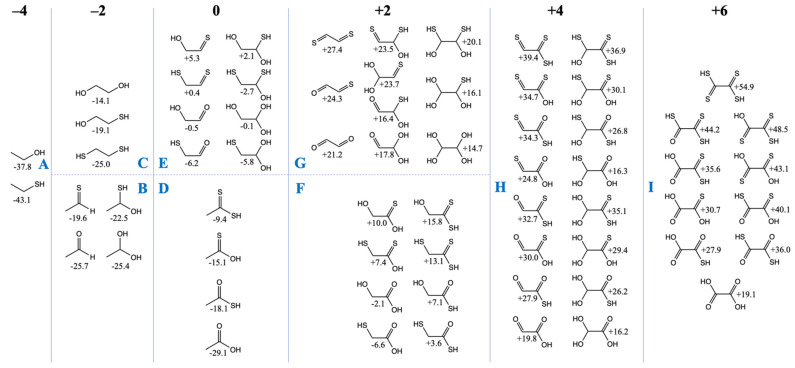
Free Energies of C_2_ CHOS compounds by oxidation groups (*G_r_*_0_ values in kcal/mol).

We now turn our attention to the C_2_ compounds of CHOS and compare them to their CHO counterparts. Do the same trends we’ve seen for the C_1_ compounds hold in the C_2_ cases? In Figure 2, we have grouped the compounds according to the total formal oxidation state of the carbons, e.g., ethanethiol (CH_3_CH_2_SH) has six hydrogens (+1 each) and one sulfur (−2), and thus the carbons must add up to −4 for an overall neutral molecule.

Similar to our more extensive study of CHO compounds [6], *G_r_*_0_ values are lowest for the most reduced compounds and *G_r_*_0_ values increase with oxidation. All compounds in the −4 and −2 oxidation groups have negative *G_r_*_0_ values, i.e., they are more stable relative to the reference reactants CO_2_, H_2_ and H_2_S. Similar to the C_1_ case, thiol groups are favored over alcohols. In Figure 2A, ethanethiol is more stable than ethanol by ~5 kcal/mol, and in Figure 2C, replacing an OH by an SH is favorable by 5–6 kcal/mol. In Figure 2B, ethanal is 6 kcal/mol more stable than its counterpart with a C=S thione group (unlike the large gap of 13 kcal/mol in the C_1_ case). For the C_2_ case, hydrating the aldehyde hardly changes its *G_r_*_0_ value, while hydrating the thione stabilizes it by ~3 kcal/mol.

In the CHO compounds from our previous study [6], having a carbonyl group was always more stable than having two separate alcohol groups (on different carbons) by a significant amount (over 10 kcal). However, this trend is reversed with sulfur; having two separate thiol groups (on different carbons) is more stable than the thione (with its weaker C=S pi-bond). For the −2 oxidation group, this leads to the most stable compounds in Figure 2B (the aldehyde and its hydrate) having a similar *G_r_*_0_ value to the most stable compound (the dithiol) in Figure 2C.

For the zero oxidation group, the acids (Figure 2D) are significantly more stable than their isomers (Figure 2E) which have separate C=X and C–XH groups. In Figure 2D, the trend is similar to the C_1_ compounds: the carboxylic acid is more stable than the thioacid by 10 kcal/mol, and the thioacid is more stable than its thione isomer by 3 kcal/mol. (The CSSH compound is further destabilized by ~6 kcal/mol.) In Figure 2E, the most stable compound is mercaptoacetaldehyde (*G_r_*_0_ = −6.2 kcal/mol), as expected, because thiols have lower *G_r_*_0_ values than alcohols. Glycolaldehyde (*G_r_*_0_ = −0.5 kcal/mol) is close in stability to its sulfur counterpart (*G_r_*_0_ = +0.4 kcal/mol) because thiol stabilization over the alcohol is almost equally balanced by carbonyl stabilization over the thione. Hydration trends are similar to what we saw in Figure 2B.

The two sets of compounds in the +2 oxidation group are glycolic acid with its sulfur analogs in Figure 2F, and glyoxal with its sulfur analogs in Figure 2G. The mercaptoacid (*G_r_*_0_ = −6.6 kcal/mol) is the most stable, followed by glycolic acid (*G_r_*_0_ = −2.1 kcal). These are the only two compounds with negative *G_r_*_0_ values in this group. Overall trends comparing the substitution of oxygen with sulfur are similar to previous cases, although we note that the gap between the thiol versus alcohol is now only 3–4 kcal/mol (instead of 5–6 kcal/mol). In Figure 2G, the gap between a thione and aldehyde has also reduced further to ~3 kcal/mol.

In Figure 2G (the +4 oxidation group), energy trends are similar to previous cases both for hydration reactions and for O to S substitutions in functional groups. There are two exceptions: (OH)_2_CHCSSH (*G_r_*_0_ = +35.1 kcal/mol) is ~3 kcal/mol higher than expected from the general trend; and S=CC(=O)SH (*G_r_*_0_ = +34.3 kcal/mol) is ~4 kcal/mol higher than expected from the general trend. It is unclear why this is so, but we do not expect these sulfur analogs to play an important role given their very positive *G_r_*_0_ values. The most stable compounds in this group are the glyoxylic acid hydrate (*G_r_*_0_ = +16.2 kcal/mol) and its thione hydrate counterpart *G_r_*_0_ = +16.3 kcal/mol). Glyoxylic acid is an activated species in proto-metabolism, as discussed in our previous work [6], and not surprisingly is used (as glyoxylate) experimentally to drive proto-metabolic reactions in prebiotic chemistry.

Oxalic acid (*G_r_*_0_ = +19.1 kcal/mol) is the most stable compound in the +6 oxidation group (Figure 2I). All its sulfur counterparts have very positive *G_r_*_0_ values and they are not expected to be accessible or utilized in a sulfur-containing proto-metabolism.

### 3.3. Thermodynamics of C–S Coupling Reactions

Now that we have a lay of the land with our preliminary map of *G_r_*_0_ values for C_1_ and C_2_ CHOS compounds, we can begin to assess the thermodynamics of forming C–S bonds if these are to play a role in proto-metabolic reactions.

In a prebiotic setting where CO_2_ is reduced by a mixture of H_2_ and H_2_S, two CHOS C_1_ compounds that we might expect to see are methanethiol (CH_3_SH) and the thione-hydrate CH_2_(OH)(SH). We also expect the CHO compounds methanol, formaldehyde (and its hydrate), and formic acid to be present. (Methane, the most thermodynamically favorable product, will also be present but is unlikely to react any further in a reducing environment and can be considered a “waste” molecule.) In our previous work on formaldehyde oligomerization [11], polyols and oxanes are produced in condensation reactions forming new C–O bonds. These polyols and oxanes are marginally unfavorable thermodynamically compared to the monomer (hydrate) but the free energy difference is very small. How does forming new C–S bonds fare? 

**Figure 3:**
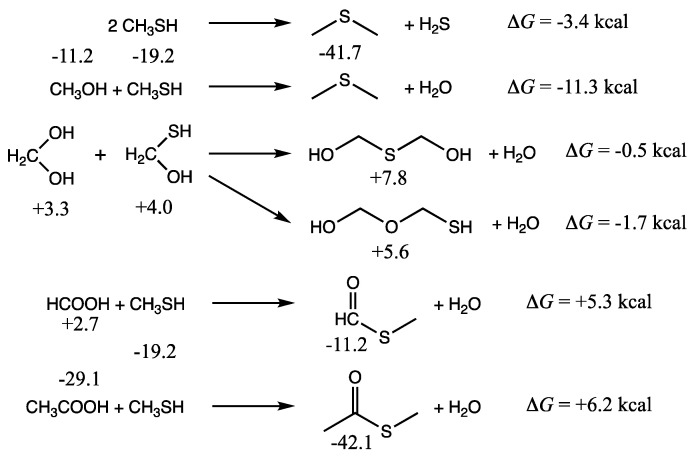
Selected C–S bond forming reactions, *G_r_*_0_ values next to structures are in kcal/mol.

As shown in the first two reactions of Figure 3, the formation of dimethylsulfide from methanethiol is exergonic. We calculate Δ*G* of the reaction by subtracting *G_r_*_0_ of the reactants from *G_r_*_0_ of the products. (Recall that reference molecules have zero *G_r_*_0_ values.) Thus, Δ*G* = (−41.7 + 0.0) − 2(−19.2) = −3.4 kcal. The condensation of methanethiol and methanol to form dimethylsulfide is more exergonic: Δ*G* = (−41.7 + 0.0) − (−11.2 + (−19.2)) = −11.3 kcal. It is certainly more favorable than forming dimethylether (Δ*G* = +5.5 kcal). Thus, we expect dialkylsulfides to be formed if methylsulfide is present.

If hydrated formaldehyde and its counterpart C_1_ thione are present, their condensation reactions are mildly exergonic, and so we might expect to see HO–CH_2_–X–CH_2_–XH compounds (X = O or S) as shown in the middle set of reactions in Figure 3. Forming the C–O–C compound is marginally more favorable than the C–S–C in this case. Thus, one might expect to see mixed polyol/polythiols depending on the concentrations of monomers. In an aqueous solution, the equilibrium will shift towards hydrolysis back into the monomers. For a 1 M solution, where water molecules outnumber solutes by 55:1, the correction factor is 2.4 kcal/mol in favor of hydrolysis [13]. We expect that for dilute solutions, monomers will be favored over condensation reactions that form C–X–C bonds (while releasing water), and hence we have not pursued calculating the free energies of polythiols or thiolanes. For the energetics of oxane/polyol formation from formaldehyde, the reader can refer to our previous work [11].

The final pair of reactions in Figure 3 illustrate thioester formation from the reaction of CH_3_SH with formic or acetic acid. These reactions are endergonic by 5.3 and 6.2 kcal respectively. In contrast, as we saw in the previous two sections, thioacid formation is endergonic by ~10 kcal. Since compounds with thiol groups are thermodynamically favored over their alcohol counterparts, and carboxylic acid groups (if they can be formed) are the most stable compounds in an oxidation group, this hints towards the role of thioesters in a prebiotic milieu as an important intermediate in chemical processes that couple endergonic and exergonic reactions.

**Figure 4:**
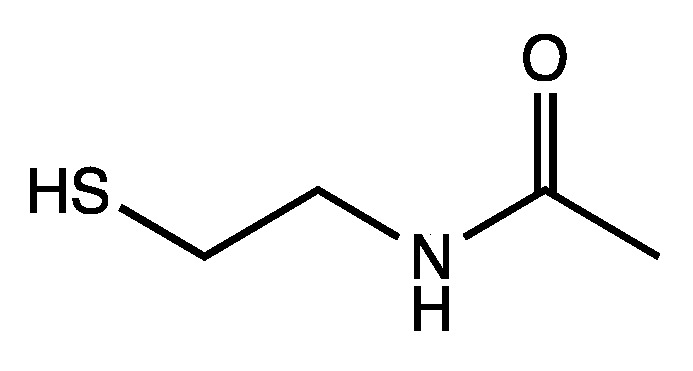
Small molecule analog of CoA.

In extant biochemical reactions involving the coenzyme CoA, forming the thioester is typically ~7 kcal uphill. Using the small molecule analog shown in Figure 4, we calculate that its condensation with acetic acid and succinic acid are +6.9 kcal and +7.5 kcal respectively. Thus, exergonic hydrolysis of such thioesters can potentially be coupled to proto-metabolite C–C bond formation where the carboxylation reactions are endergonic by 4–7 kcal, as shown in our previous work on CHO systems [6]. Our preliminary results, while promising, would not do justice to the complexity of the system, and we expect to provide a detailed examination of the connection between thioesters and potential CHO proto-metabolic systems in a future publication.

### 3.4. Sulfur Analogs of the Formose Reaction

In extant biochemical cycles, the reduction of CO_2_ to build biomass can proceed through cycles analogous to the reverse TCA cycle. We explored the thermodynamics of four such cycles in CHO systems in our previous work [6], the most interesting being the 3-hydroxypropionate/4-hydroxybutyrate (3HP/4HB) cycle because it does not involve CHO compounds with more than four carbons and avoids forming the less stable oxaloacetate. In that work, we proposed alternative pathways that could be thermodynamically more feasible than the 3HP/4HB cycle thereby avoiding some of the more challenging kinetic barriers, but we also noted that in the absence of (specialized) enzyme catalysts there would still be kinetically unfeasible steps in a prebiotic milieu.

There is a known autocatalytic reaction that builds up progressively larger CHO compounds from a C_1_ species—the formose reaction [7]. It takes advantage of aldol reactions to form new C–C bonds, and autocatalysis is aided by a retro-aldol transformation of a C_4_ species into two C_2_ compounds. It is thus analogous to the 3HP/4HB cycle, but much simpler because it avoids redox reactions: formaldehyde is the C_1_ ‘food’ species, glycolaldehyde is the linchpin C_2_ species, and all compounds involved remain in the zero oxidation group. In contrast for the 3HP/4HB cycle, while acetate (the C_2_ linchpin) is in the zero oxidation group, CO_2_ (+4 oxidation group) is the C_1_ food species and therefore reducing equivalents of H_2_ are required for the cycle to be realized.

**Figure 5:**
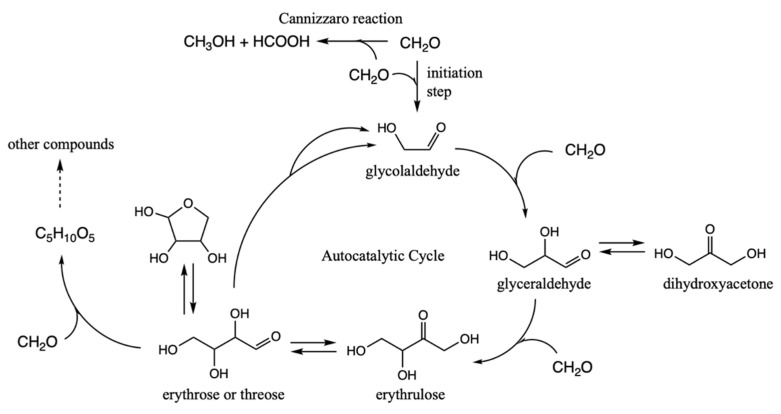
Key features of the formose reaction autocatalytic cycle.

The problem with the formose reaction is that it is a mess [8], and a slew of compounds are formed in an essentially uncontrolled reaction. Could the presence of sulfur introduce some form of thermodynamic control to the reaction? How might the kinetics change? Is there a path towards taming the formose reaction as a stepping stone towards proto-metabolic cycles that more closely resemble what extant life uses? Building on what we have learned from our survey of CHOS C_1_ and C_2_ compounds described earlier, this subsection presents our free energy map of sulfur analogs to the formose reaction. A brief summary of the key compounds in the (non-sulfur-containing) formose reaction are shown in Figure 5.

In discussing the results, we will repeatedly make reference to our earlier free energy map of the thermodynamics and kinetics of the formose reaction (up to C_4_); this paragraph provides the highlights from that work [11]. Forming glycoaldehyde directly from CH_2_O is very challenging kinetically. We previously calculated the barrier for direct dimerization to be 45.3 kcal. Experimentally, in a solution only containing CH_2_O, there is a long induction period. However once even a small amount of glycolaldehyde is formed (or added to the solution as an initiator), the reaction proceeds rapidly producing a wide variety of sugars, mostly in the C_4_ to C_7_ range. With C_2_ present, the difficult C_1_ + C_1_ → C_2_ reaction is bypassed by the much lower barrier C_2_ + C_1_ → C_3_ and C_3_ + C_1_ → C_4_ reactions. The retro-aldol C_4_ → C_2_ + C_2_ reaction regenerates (more) C_2_ and accelerates the consumption of C_1_ making the cycle autocatalytic. CH_2_O can also form polyols and oxanes but hydrolysis in an aqueous solution favors re-forming the monomer. On the other hand, the Cannizzaro disproportionation reaction parasitizes the cycle (to be discussed in a later subsection of this paper). Extensive documentation of experimental results on the formose reaction can be found in a long article by Mizuno and Weiss [27].

If H_2_S was present as a source of sulfur, one might expect a starting mixture of the hydrates CH_2_(OH)_2_ and CH_2_(SH)(OH) in aqueous solution, as they are relatively close in energy with *G_r_*_0_ values of +3.3 and +4.0 kcal respectively. Our calculated barrier for the direct C–C coupling reaction of CH_2_O and CH_2_S is 26.0 kcal, which is much lower than 45.3 kcal for the direct dimerization of CH_2_O, but recall from Figure 1 that CH_2_S is 13.3 kcal/mol less stable than CH_2_O, which accounts for two-thirds of the difference. Mercaptoacetaldehyde (*G_r_*_0_ = −6.2 kcal) is the C_2_ species formed, and the reaction is thermodynamically favorable (Δ*G* = −19.7 kcal from the hydrates). Since a range of C_1_ and larger species (C_2_, C_3_, etc.) are observed experimentally in prebiotic reactions [28,29,30,31,32,33] by reducing CO_2_ (or bicarbonate or CO or COS) simulating hydrothermal vent prebiotic chemistry, and since the C_1_ + C_1_ → C_2_ initiation step is not important for the cycle, we need not worry about the initiation step. Our starting point will be the C_2_ linchpin species, mercaptoacetaldehyde, the thiol analog of glycolaldehyde. Mercaptoacetaldehyde has also been proposed as central in prebiotic scenarios involving the amino acid cysteine [34].

**Figure 6:**
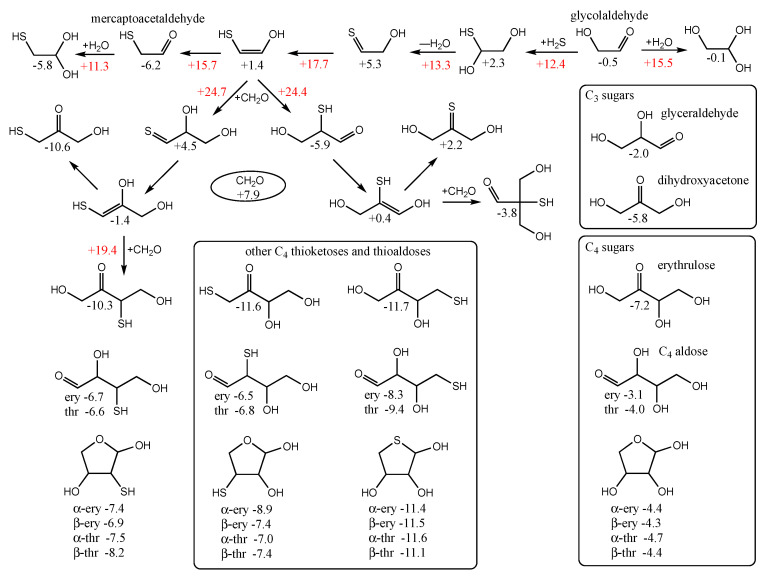
Selected C_2_ to C_4_ thermodynamics and kinetics of thioaldoses and thioketoses, *G_r_*_0_ values next to structures and arrows are in kcal/mol.

Since *G_r_*_0_ = −6.2 kcal/mol for mercaptoacetaldehyde, it is favorable thermodynamically to be (one among many possible compounds) produced prebiotically from a source containing CO_2_, H_2_ and H_2_S. (It may not be as easily observed experimentally because it participates in further reactions.) Mercaptoacetaldehyde can also potentially be formed from glycolaldehyde in the presence of H_2_S as shown in the top row of Figure 6. The reaction is overall thermodynamically favorable, Δ*G* = −6.2 − (−0.5) = −5.7 kcal. Note that the cis-enol of mercaptoacetaldehyde as shown in Figure 6 is more stable than the trans-enol (not shown) by ~2 kcal/mol in our calculation of *G_r_*_0_.

By calculating the energies of the transition states (*G_r_*_0_ values in red next to arrows), we can estimate the reaction kinetics. For example, the first step of adding H_2_S to glycolaldehyde has a barrier of +12.4 − (−0.5) = 12.9 kcal. (The corresponding dehydration barrier in the reverse reaction is +12.4 − (+2.3) = 10.1 kcal.) The calculated stepwise barriers for the overall transformation of glycolaldehyde to mercaptoacetaldehyde (involving formation of the thione intermediate and its enol) are in the 11–14 kcal range. (At both ends on the top row, we also show the hydration reactions of mercaptoacetaldehyde and glycolaldehyde for completeness; see Supplementary Materials for transition state structures.)

**Figure 7:**
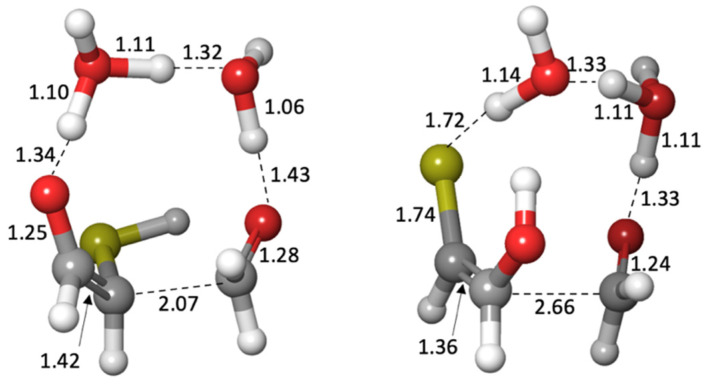
Transition states for the aldol addition of CH_2_O to mercaptoacetaldehyde. Bond distances shown are in Å.

In our previous work on CH_2_O oligomerization [11], aldol additions of CH_2_O proceed via the enol. We see the same for mercaptoacetaldehyde, except that its asymmetry allows for two possible products: the less favorable thione (*G_r_*_0_ = +4.5 kcal) and the more favorable aldehyde (*G_r_*_0_ = −5.9 kcal) that has a thiol on the central carbon. Kinetically, we might also expect the aldehyde to be favored because the thiol carbon of the enol is a better nucleophile than the alcohol carbon. However, our calculated barriers are essentially identical; this is after optimizing multiple transition states and the lowest energy structures are shown in Figure 7.

Considering the enol (*G_r_*_0_ = +1.4 kcal) and CH_2_O (*G_r_*_0_ = +7.9 kcal) as the reactants, the barrier to forming the C_3_ aldehyde is 24.4 − (1.4 + 7.9) = 13.3 kcal, and the barrier to the C_3_ thione is 24.7 − (1.4 + 7.9) = 13.6 kcal. If mercaptoacetaldehyde and CH_2_O were the reactants, the barriers would, respectively, be 24.4 − (−6.2 + 7.9) = 22.7 kcal and 24.7 − (−6.2 + 7.9) = 23.0 kcal. These calculated barriers are very similar to our previous work for the C_1_ + C_2_ → C_3_ aldol addition of glycolaldehyde and CH_2_O of 22.3 kcal (or 13.0 kcal from the enol). Thus, in a mixture that contained glycolaldehyde, mercaptoacetaldehyde, and CH_2_O, the kinetics for this first aldol addition (C_1_ + C_2_ → C_3_) are similar and both C_2_ “reactants” will consume the C_1_ food source (CH_2_O) at similar rates.

Let us now consider the thermodynamics of the C_1_ + C_2_ aldol addition. In the CHO system, forming glyceraldehyde (*G_r_*_0_ = −2.0 kcal) is favorable with Δ*G* = −2.0 −(−0.5 + 7.9) = −9.4 kcal. The analogous reaction in the CHOS system with mercaptoacetaldehyde, forming the C_3_ aldehyde-thiol, is similarly favorable: Δ*G* = −5.9 − (−6.2 + 7.9) = −7.6 kcal. On the other hand, forming the thione-diol is slightly unfavorable: Δ*G* = +4.5 − (−6.2 + 7.9) = +2.8 kcal. Since the C_3_ aldehyde-thiol has the lower *G_r_*_0_ value, it could be thermodynamically favored over glyceraldehyde in an equilibrating mixture with multiple reactants.

However, the situation is more complicated because “globally” among the C_3_ structures, the thioketose (*G_r_*_0_ = −10.6 kcal, leftmost structure in the second row of Figure 6) is the most stable, and access to it via enolization comes from the less thermodynamically favorable aldol addition. The intermediate enol with a terminal thiol (*G_r_*_0_ = −1.4 kcal, leftmost structure in the third row of Figure 6) is also the starting point for further aldol addition of CH_2_O to form the linear C_4_ thiosugars. On the other hand, the enol of the C_3_ aldehyde-thiol would lead to a branched C_4_ thiosugar (*G_r_*_0_ = −3.8 kcal), assuming our earlier argument that the thiol carbon of the enol is the better nucleophile. However, as we saw for C_1_ + C_2_ → C_3_, addition to the alcohol side of the enol is just as viable kinetically, and likely more so in this case to avoid steric hindrance. Thus, access to the linear C_4_ thio-sugars is possible through both branches. What role might the C_3_ thioketose play? Analogous to dihydroxyacetone, as discussed in our previous work [6], it may be an “off-cycle” compound that forms an equilibrating pool of inter-connected compounds [35] that could stabilize the cycle and provide a form (albeit simple) of regulatory control. (Dehydrations of C_3_ sugars may also be a part of this pool; see Supporting Materials).

The C_1_ + C_3_ addition to form the C_4_ thioketose (*G_r_*_0_ = −10.3 kcal/mol, left side of Figure 6) is thermodynamically favorable with Δ*G* = −10.3 − (−10.6 + 7.9) = −7.4 kcal, very similar to the aforementioned C_1_ + C_2_ → C_3_ addition of Δ*G* = −7.6 kcal. The barrier for the C_1_ + C_3_ → C_4_ aldol addition is 19.4 − (1.4 + 7.9) = 8.1 kcal from the enol, noticeably lower than 13.3 kcal in the analogous C_1_ + C_2_ → C_3_. In our previous work on the CHO system [11] (leading to erythrulose), the barrier is 8.5 kcal for C_1_ + C_3_ → C_4_, which is similarly lower than the 13.0 kcal barrier for C_1_ + C_2_ → C_3_. Thus, kinetically, we expect the CHOS analog of the formose reaction to show similar behavior as the CHO system under appropriate experimental conditions that facilitate the reaction. Thermodynamically (left side of Figure 6), the thioketose is ~4 kcal more stable than its open-chain thioaldoses, while the ring structures are ~1 kcal more stable than the open thioaldoses. Once again, this is similar to the non-sulfur analogs (bottom right box in Figure 6) of erythrulose, erythrose, threose, and the ring structures. We can think of the ketose, the open chain aldose, and the furanose as an equilibrating pool of compounds.

For the C_4_ sugars, the 3-thioketose turns out to be marginally less stable than both the 1-thioketose and 4-thioketose that have terminal thiols (Figure 6, central lower box). As for the aldoses, the 3-thioaldose and 2-thioaldose have similar energies, while the 4-thioaldose with its terminal thiol is the most stable. This is also true for the ring structures, and interestingly the 4-thioaldose rings (*G_r_*_0_ values of −11.1 to −11.6 kcal/mol) are similar in stability to the 4-thioketose (*G_r_*_0_ = −11.7 kcal/mol). This suggests that a possible role played by (terminal) thiol groups in a prebiotic setting is to stabilize the corresponding aldose rings.

A key autocatalytic step in the formose reaction is the retroaldol reaction of the C_4_ aldose back into two C_2_ linchpin molecules. In the CHO system, this reaction starting from threose is marginally uphill with Δ*G* = 2(−0.5) − (−4.0) = +3.0 kcal. In the sulfur analog, the thioaldose splits into mercaptoacetaldehyde and glycolaldehyde. For 4-thiothreose, we see a similar result: Δ*G* = (−0.5) + (−6.2) − (−9.4) = +2.7 kcal. However, for 3-thiothreose, the reaction is now energetically neutral with Δ*G* = (−0.5) + (−6.2) − (−6.6) = −0.1 kcal. (2-thiothreose shows a similar result with Δ*G* = +0.1 kcal). Thus, considering only the C_4_ species for the moment, we might expect over time a depletion of the 2- and 3-thio-sugars, and possible accumulation of the 4-thiosugars, favoring the aldose rings that are more resistant to hydrolysis. The reality would be a lot messier with other aldol and retro-aldol reactions occurring, alongside Cannizzaro side-reactions.

Stepping back to look at the overall thermodynamic map, we see that the C_3_ and C_4_ species show similar trends as the C_1_ and C_2_ species discussed earlier. Compared to the reference compounds, thiol groups are favored over their alcohol counterparts and are most stable in the terminal position. Thiones with their weaker C=S bonds are less stable than their carbonyl counterparts. We also have preliminary data (for a future publication) showing that the trends for sulfur analogs for the larger molecular acids mirror those for we previously discussed for the smaller molecules. Overall, we see many similarities for both the thermodynamics and kinetics when comparing individual steps in the formose reaction of the CHO system to its sulfur analogs.

### 3.5. Can Dithiol Groups Influence Sugar Formation?

Could having a thiol group in a sugar make a relevant and interesting difference? One possibility we explore in this subsection is based on the experimental work of Eschenmoser and colleagues [36], where they found that starting with glycolaldehyde-2-phosphate and formaldehyde led to a higher yield of ribose among the pentose-2,4-diphosphates formed. If phosphate can “direct” the reaction to favor certain products over others (in a messy formose-like reaction), can sulfur do the same? If sulfur was primordial to phosphate in prebiotic systems, could it have played an analogous role?

**Figure 8:**
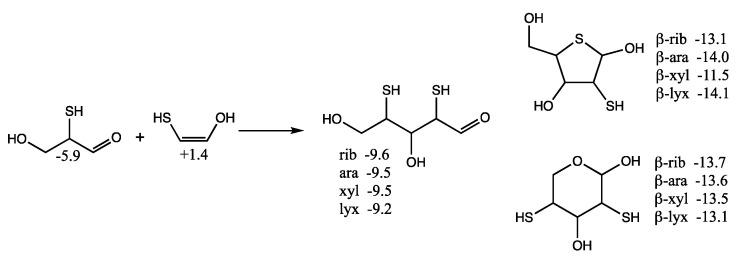
Formation of C_5_ 2,4-dithioaldoses from aldol C_2_ + C_3_ reaction; *G_r_*_0_ values next to structures are in kcal/mol.

Considering mercaptoacetaldehyde as the sulfur analog of glycolaldehyde-2-phosphate, in the presence of formaldehyde we expect aldol addition to favorably form the C_3_ aldehyde-thiol (as discussed in the previous section), i.e., the analog of glyceraldehyde-2-phosphate. Aldol addition of mercaptoacetaldehyde (via its enol) with the C_3_ aldehyde-thiol leads to 2,4-dithioaldoses (the sulfur analogs of the C_5_ aldose-2,4-diphosphates) as shown in Figure 8. The rings are more stable than the open chain structures. Unlike the CHO sugars, the pyranoses are not more stable than the furanoses but have comparable free energies. This is because having sulfur in the ring provides a 2–3 kcal/mol stabilization (as seen for the thiotetroses in Figure 6).

For the open chain pentoses, our calculated *G_r_*_0_ values have ribose being the most stable followed by arabinose, xylose, lyxose. However, the difference in free energy is tiny and certainly within the computational error; we cannot claim that incorporation of sulfur favors ribose over the other aldopentoses. For the β-pyranoses, we see the same order of stability as the open chain structures, and again the differences are tiny and within the computational error. For the β-furanoses, arabinose and lyxose have lower *G_r_*_0_ values than ribose with xylose being the least stable. We have no explanation why this is or if this is some artifact of the calculation (possibly not finding the best conformers in some cases).

**Figure 9:**
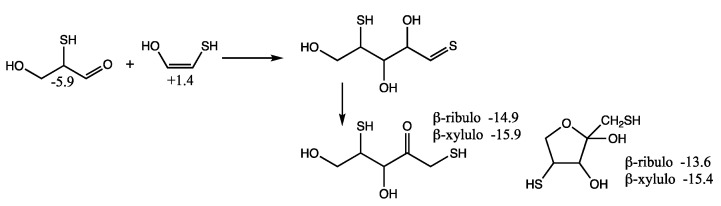
Formation of C_5_ 1,4-dithio-2-ketoses from aldol C_2_ + C_3_ reaction; *G_r_*_0_ values next to structures are in kcal/mol.

Although we expect the C_2_ + C_3_ addition to form the C_5_ 2,4-dithioaldoses to be kinetically favored (because the thiol carbon of the enol is more nucleophilic than the alcohol carbon), we also consider the possibility of forming 1,4-dithio-2-ketopentose since it leads to more thermodynamically favored ketoses as shown in Figure 9. (We have switched the position of the OH and SH in the enol in Figure 9 compared to Figure 8 to make the aldol addition clearer.) The thione intermediate formed is likely to isomerize to the more favored keto form. Our calculated *G_r_*_0_ values have the open chain sulfur analog of xylulose more stable than the ribulose by 1 kcal/mol. The furanoses are not as stable as the open chain structures.

**Figure 10:**
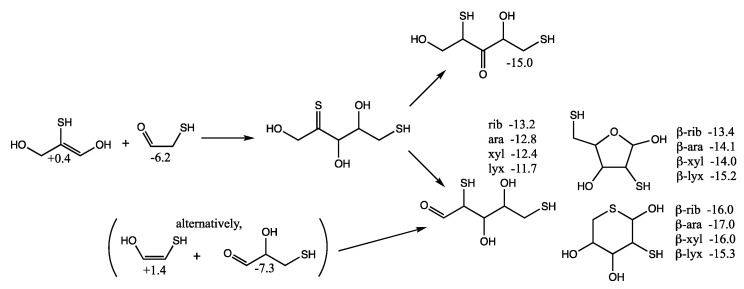
Formation of C_5_ 1,4-dithio-3-ketoses and 2,5-dithioaldoses from aldol C_2_ + C_3_ reaction; *G_r_*_0_ values next to structures are in kcal/mol.

However, there is another possibility. If C_3_ acts as the enol (rather than the C_2_), a C_5_ thione intermediate can be formed that could subsequently isomerize into a 1,4-dithio-3-ketose or a 2,5-dithioaldose, as shown in Figure 10. The most sTable 3-ketose has *G_r_*_0_ = −15.0 kcal/mol (its diastereomer is only 0.3 kcal less stable). For the open chain 2,5-dithioaldoses, the ribose analog is the most stable followed by arabinose, xylose, and lyxose. The furanoses, with a pendant thiol in the 5-position are slightly more stable than the open chain structures. The pyranoses with sulfur in the ring are the most stable group. For the ring structures, we have no explanation for the relative ordering of the most stable different stereoisomers according to our calculated *G_r_*_0_ values.

An alternative route (see parenthesis in Figure 10) to the C_5_ 2,5-dithioaldoses is the aldol addition of the C_2_ enol with 3-thioglyceraldehyde (*G_r_*_0_ = −7.3 kcal/mol), the isomer of the C_3_ ketone (the most stable C_3_ sulfur analog in Figure 6 with *G_r_*_0_ = −10.6 kcal/mol). If thiols could be precursors to phosphates in a prebiotic world, the 2,5-dithioribose analog could be a stand-in for ribose-2,5-diphosphate.

There are other possible products from aldol additions of these sulfur analogs that we have not discussed. (See Supplementary Materials). For example, in the C_2_ + C_3_ → C_5_ addition, one of the species may not contain sulfur, and this would lead to a range of sugars with just one thiol group rather than two. As a second example, we have not discussed the C_4_ + C_1_ → C_5_ addition, which would lead to other isomers such as 3,5-dithio-2-ketoses, 1,3-dithio-2-ketoses and 3,5-dithioaldoses. Furthermore, we have mainly focused on non-branched sugars, and we have only shown one example, the branched tetrose in Figure 6. We expect these thermodynamically less stable branched sugars to be less prevalent than their straight-chain counterparts.

Our limited foray into sulfur analogs of the formose reaction is clearly not exhaustive. Our goal here is to provide a flavor of the myriad possible reactions, intermediates, and products, in this system. Based on our limited analyses, we can draw some general conclusions. Substituting an alcohol with a thiol group is thermodynamically favorable. Thiol groups in the terminal position are particularly favored. Sulfur in the sugar ring is thermodynamically favored. Furthermore, the presence of sulfur provides some asymmetry to the aldol addition reactions, and the lower electronegativity of sulfur means that in an enol, the thiol carbon is a better nucleophile which may provide some “directing” ability that favors some subsets of products over others.

While we have speculated about the possibility that thiols might be precursors to phosphates in aldol reactions of sugars, our results thus far are inconclusive on this topic. However, there are tantalizing analogies. In the pentose phosphate pathway, the sugars involved have terminal phosphates, and our limited study finds that terminal thiols are thermodynamically favored. By including thiols in the mix, we find that aldoses can be as thermodynamically stable as ketoses for C_4_ and C_5_, while this is not so in CHO sugars where the ketose is typically 2–3 kcal more stable than the aldose.

### 3.6. Sulfur Analogs of the Cannizzaro Reaction

As this article has focused on the sulfur analogs of the sugars in the formose reaction, we would be remiss by not (briefly) discussing the Cannizzaro side-reactions. Let us first consider the simplest case involving monomeric formaldehyde. The reaction CH_2_O + CH_2_(OH)_2_ → CH_3_OH + HCOOH is thermodynamically favorable (Δ*G* = −20.7 kcal) and kinetically favorable (barrier of 20.3 kcal) [11], and certainly outcompetes the dimerization of CH_2_O to glycolaldehyde. It is also a disproportionation reaction, converting the zero-oxidation formaldehyde into reduced and oxidized products (−2 for methanol and +2 for formic acid respectively). These reactions are why the formose reaction is messy and the products include a range of alcohols and carboxylic acids (or carboxylates in the alkaline solution used experimentally) [8]. Does the presence of sulfur analogs make a difference?

In the presence of H_2_S, we expect some amount of CH_2_(SH)(OH) to be present in solution as a starting point. Using the *G_r_*_0_ values from Figure 1, we can consider the following possibilities.

CH_2_O + CH_2_(SH)(OH) → CH_3_SH + HC(=O)OH Δ*G* = −28.6 kcal

CH_2_O + CH_2_(SH)(OH) → CH_3_OH + HC(=O)SH Δ*G* = −10.3 kcal

Based on the trends discussed earlier—thiols are more stable than alcohols, and carboxylic acids are more stable than thioacids—it is no surprise that thermodynamically, methanethiol and formic acid are the preferred products. Our calculated barrier is 21.5 kcal, not too different from the non-sulfur Cannizzaro reaction.

**Figure 11:**
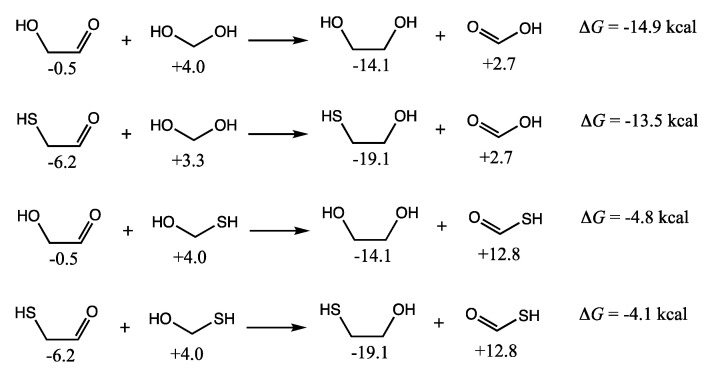
C_2_ + C_1_ disproportionation reactions (*G_r_*_0_ values next to structures in kcal/mol).

C_2_ compounds in the mixture could also undergo disproportionation. The four main possibilities involving a C_1_ and a C_2_ species are shown in Figure 11; we have not included higher-energy starting reactants that are unlikely to be present in the mixture such as H_2_C=S (which would undoubtedly lead to significantly exergonic reactions as a highly activated species). The disproportionation of glycolaldehyde with (hydrated) formaldehyde leading to ethylene glycol and formic acid is exergonic by 14.9 kcal. Not too different is the disproportionation of mercaptoacetaldehyde with formaldehyde leading to mercaptoethanol (*G_r_*_0_ = −19.1 kcal) and formic acid (Δ*G* = −13.5 kcal). Reactions with CH_2_(SH)(OH) leading to the thioacid are still exergonic but less favorable.

Mercaptoethanol could play an important role as a precursor to CoA. For example, the condensation of mercaptoethanol with acetamide leads to the CoA analog shown in Figure 4. Cannizzaro disproportionation reactions such as those shown in Figure 11 could be a source for mercaptoethanol. One could imagine a range of other sulfur-containing molecules produced by disproportionation, which we have not catalogued in the present work, but we do expect the trends in free energy to be similar to what we have analyzed thus far.

## 4. Conclusions and Future Work

In this article, we present a thermodynamic free energy map for the C_1_ and C_2_ compounds that may be present in a (possibly hydrothermal) prebiotic milieu where CO_2_, H_2_, H_2_S are present as reactants. We find that thiols are significantly more stable than alcohols, while thiones are significantly less stable than carbonyls. However, hydrated thiones are only marginally less stable than their aldehyde counterparts leaving the door open to the possibility that thiones could still play a role as reaction intermediates. Based on our calculated *G_r_*_0_ values, we expect (thermodynamically favored) CH_3_SH to be present and participate in reactions to form dialkylsulfides.

Our main focus was tracing the key reactions of CHOS compounds in the smallest autocatalytic cycle of the formose reaction. Mercaptoacetaldehyde is the linchpin C_2_ species that plays the role analogous to glycolaldehyde. The asymmetry of the mercaptoacetaldehyde enol (and by extension other related enols) potentially favors some aldol additions over others, opening the possibility to different kinetic and/or thermodynamic controls, which could lead to reaction channels that favor some reaction intermediates and products over others. Thiols are favored in the terminal positions of the sugar, possibly analogous to what we see in the sugar phosphates of extant biochemistry. Sulfur in ring structures of sugars also shift the aldehyde-ketone equilibrium away from exclusively favoring the ketoses over the aldoses.

Cannizzaro reactions may produce both carboxylic acids and thioacids, along with thiols and thio-alcohols such as mercaptoethanol. The presence of these substances could lead to a rich world of thioester chemistry, and these compounds could further participate in autocatalytic cycles that provide the beginnings of a proto-metabolic chemistry. Although thioacids are ~10 kcal less stable than their carboxylic acid counterparts, thioesters are 5–7 kcal less stable than their hydrolysis products of carboxylic acid and thiol. Hydrolysis of thioesters could help drive the carboxylation reactions needed to build CHO compounds in autocatalytic cycles. In this article, we did not further discuss these reactions because as we embarked on exploring this chemical space, it has turned out to be far larger than we anticipated. We are actively working on filling the large gaps of a more extensive free energy map involving thioesters, and we look forward to providing those results to our readers in the near future. For an example of how thioesters and dithio compounds can be experimentally coupled in prebiotic chemistry, see [37].

Our present work provides a baseline free energy map for potential CHOS metabolites in the core, and future work includes extending this map to include nitrogen-containing compounds such as amino acids, pterins, pyrroles, and pyrimidines, that may play a role in wider intermediary metabolism. Such compounds are also expected to play a role as primitive catalysts and co-factors in the establishing of proto-metabolic cycles.

## Data Availability

The data presented in this study are available in Supplementary Materials.

## Supplementary Materials

The following are available online at https://www.mdpi.com/article/10.3390/life12111763/s1, Supplementary Materials document. (1) Reaction schemes to form dithiopentoses; (2) *G_r_*_0_ values for zero-oxidation C_3_ CHOS compounds; (3) Transition state structures for conversion of glycolaldehyde to mercaptoacetaldehyde.
