# Supplementary material for: Preliminary Free Energy Map of Prebiotic Compounds Formed from CO2, H2 and H2S
Source: Life (Basel). 2022 Nov 2;12(11):1763. doi: 10.3390/life12111763 (PMC9696716; doi:10.3390/life12111763)
Supplement: Supplementary file 1 [file life-12-01763-s001.zip › life-2006259-supplementary.pdf]

Supplementary Materials for:

Preliminary Free Energy Map of Prebiotic Compounds formed from CO_2, H_2 and H_2S

Jeremy Kua * and Nicole A. Miller
Department of Chemistry & Biochemistry, University of San Diego, San Diego, CA 92110, U.S.A.
* Correspondence: jkua@sandiego.edu (J.K.)

Part 1: Reaction schemes to form other dithiopentoses

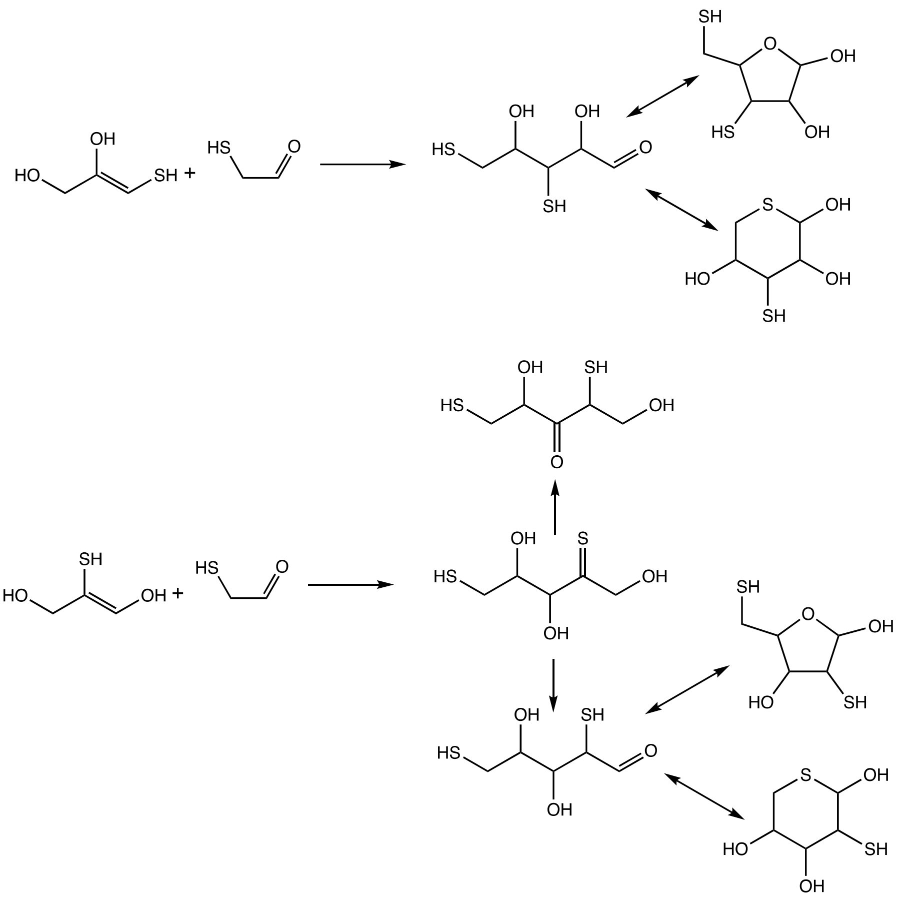

Figure S1a. C_3 enol + C_2 aldehyde aldol additions to form dithiopentoses

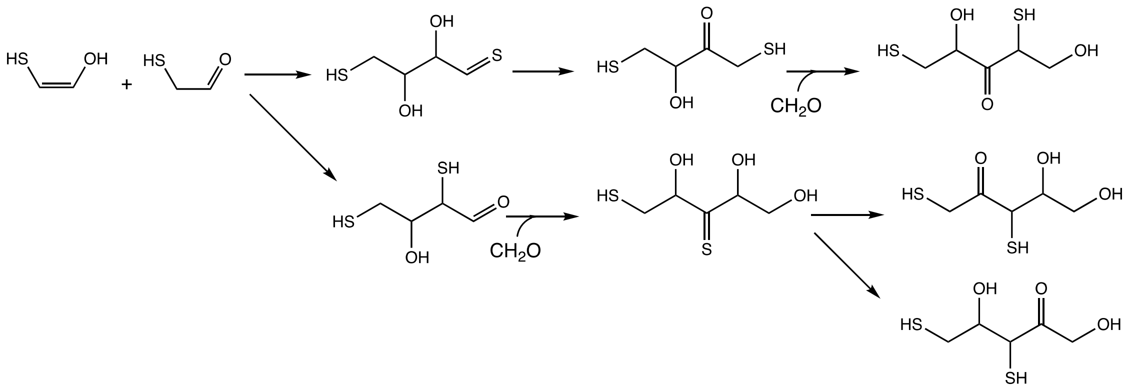

Figure S1b. Mercaptoaldehyde aldol dimerization followed by addition of CH_2O to form dithiopentoses

Part 2: G_{r0} values for zero-oxidation dehydrated C_3 compounds and its analogs.

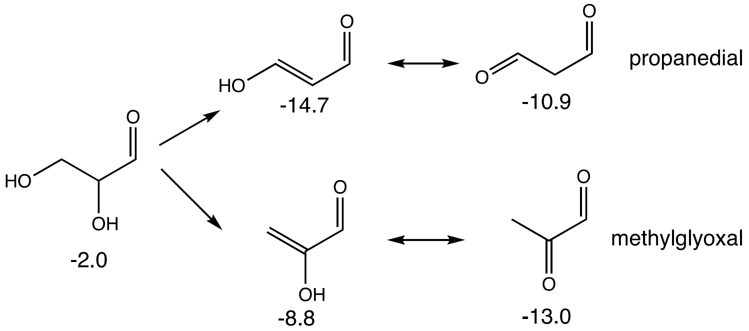

Figure S2a. Dehydration of glyceraldehyde to propanedial and methylglyoxal (G_{r0} in kcal/mol)

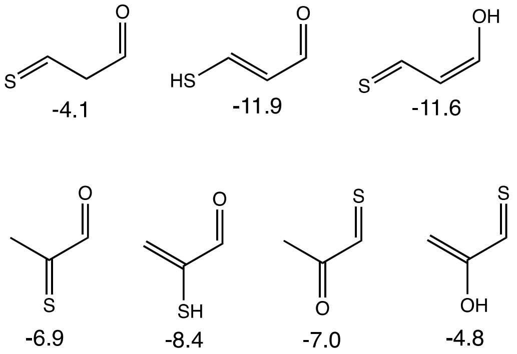

Figure S2b. Sulfur analogs of the dehydrated C_3 compounds (G_{r0} in kcal/mol)

Part 3: Additional Transition State Structures

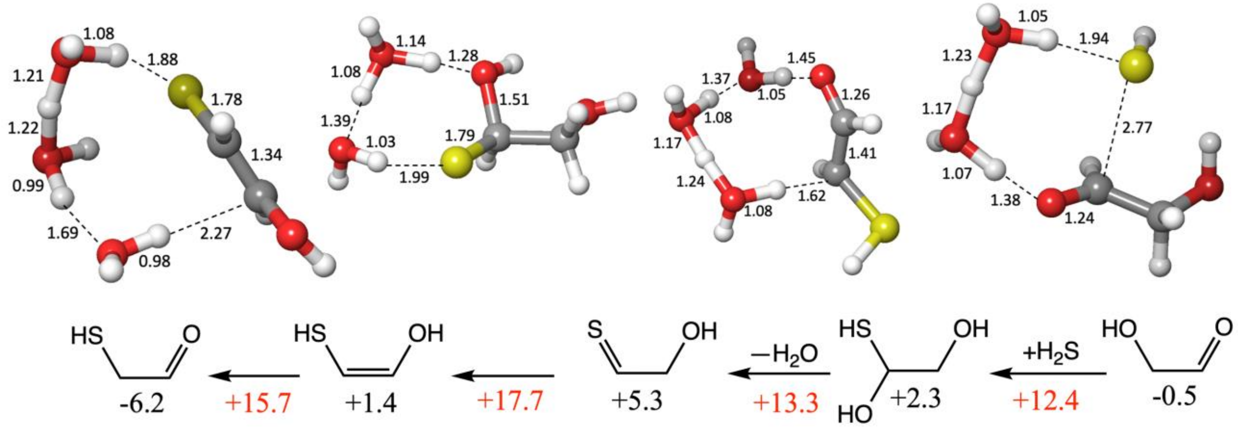

Figure S3. Transition State structures for conversion of glycolaldehyde to mercaptoacetaldehyde
